# Finite barrier bound state

**DOI:** 10.1038/s41377-024-01417-1

**Published:** 2024-03-08

**Authors:** Tao Liu, Kai Bai, Yicheng Zhang, Duanduan Wan, Yun Lai, C. T. Chan, Meng Xiao

**Affiliations:** 1 Key Laboratory of Artificial Micro- and Nano-structures of Ministry of Education and School of Physics and Technology, Wuhan University, 430072 Wuhan, China; 2 National Laboratory of Solid State Microstructures, School of Physics, and Collaborative Innovation Center of Advanced Microstructures, Nanjing University, 210093 Nanjing, China; 3 Department of Physics, The Hong Kong University of Science and Technology, Clear Water Bay, Kowloon, 999077, Hong Kong, China; 4 Wuhan Institute of Quantum Technology, 430206 Wuhan, China

**Keywords:** Photonic crystals, Nanophotonics and plasmonics

## Abstract

A boundary mode localized on one side of a finite-size lattice can tunnel to the opposite side which results in unwanted couplings. Conventional wisdom tells that the tunneling probability decays exponentially with the size of the system which thus requires many lattice sites before eventually becoming negligibly small. Here we show that the tunneling probability for some boundary modes can apparently vanish at specific wavevectors. Thus, similar to bound states in the continuum, a boundary mode can be completely trapped within very few lattice sites where the bulk bandgap is not even well-defined. More intriguingly, the number of trapped states equals the number of lattice sites along the normal direction of the boundary. We provide two configurations and validate the existence of this peculiar finite barrier-bound state experimentally in a dielectric photonic crystal at microwave frequencies. Our work offers extreme flexibility in tuning the coupling between localized states and channels as well as a new mechanism that facilitates unprecedented manipulation of light.

## Introduction

**Fig. 1:**
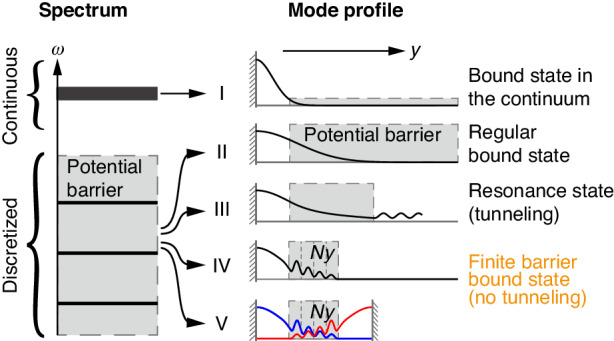
Illustration of bound state in the continuum (BIC), regular bound state, resonance state, and finite barrier bound state. Counterintuitively, as insets IV and V show, a state gets trapped completely by a bandgap material with a finite and very small thickness

The spectrum of a system typically consists of continuous spectra and discrete spectra (left panel of Fig. 1). Conventional wisdom says that the eigenvalue spectrum of bound states is discrete, while the eigenvalue spectrum of unbound states forms a continuum. For electronic systems, if the particle’s energy is lower than the potential energy at infinity, the state is bound and the corresponding energy spectrum is discrete. While the particle whose energy is higher than the potential energy is scattered and the corresponding energy spectrum is continuous. For light and sound waves, discrete states form due to the boundary condition imposed by a barrier, which is a material that forbids wave propagation (e.g., having a “bandgap”^1,2^). The discrete state can be perfectly confined by the barrier if the width of the barrier is infinite (Fig. 1-II). When the width of this barrier is finite, there is some probability that the state can tunnel through the barrier and become a resonance state (Fig. 1-III). If a state’s energy lies inside the continuous spectrum, it will unavoidably couple with states in the continuum and become a resonance state. As an exception to this rule, bound states in the continuum (BICs) can be spatially bound with energy inside the continuous spectrum (Fig. 1-I)^3–9^. Here, we show a counterintuitive concept in parallel with BICs: a state can get completely trapped (infinite Q factor if no intrinsic loss) by a bandgap material with a finite and very small thickness. The solid black line in Fig. 1-IV sketches one such state when the number of lattice sites along the normal direction of boundary is ${N}_{y}=4$ where the bulk gap is not well-defined. Later we will show that the total number of states trapped equals ${N}_{y}$, which is 4 for this case.

A state being completely trapped indicates that there is no probability for the state to tunnel through the bandgap material. Considering a symmetric double-well configuration as shown in Fig. 1-V, then a state localized on the left-hand-side well (blue line) cannot tunnel into the state on the right-hand-side well (red line). Or equivalently, there is no coupling (no hopping) between the two states in Fig. 1-V. The above discussion about trapped states can also be generalized to waveguide modes with one additional dimension. We note that the probability of tunneling is a crucial factor for quantum information processing such as controlling the lifetime of the trapped states^10,11^, manipulation of entangled states^12–14^, and non-abelian braiding of photons^15,16^. On the other hand, unwanted coupling between states introduces harmful crosstalk which limits the integration of multiple components into a compact device^17^. There is recent intense attention on topological artificial structures^18–21^, where topological boundary modes, hinge modes, corner modes, and modes trapped by topological defects such as dislocations or disclinations are robust against disorder and fabrication imperfections^22–34^. However, even these boundary and hinge modes suffer from the tunneling effect for systems with a small thickness^35–39^.

In this study, we experimentally demonstrate the existence of the bound states trapped by a finite barrier as sketched in Fig. 1-IV, V. We start with the coupling between two boundary modes localized on the opposite edges of a strip geometry of a two-dimensional (2D) photonic crystal (PC). We show that the coupling can vanish (i.e., no tunneling) at specific wavevectors for a narrow strip with very few lattice sites, which is significantly different from the prevailing understanding that the coupling vanishes only when the width of the strip is large enough. For convenience, we call these special wavevectors nodal wavevectors. We show that the number and the specific values of the nodal wavevectors can be controlled at will. When one side of the strip is opened to free space as shown in Fig. 1-IV, only one of the boundary modes remains. We find that the remaining boundary mode exhibits an infinite Q factor at the nodal wavevectors. Since the boundary mode at these nodal wavevectors has their frequencies lying in the continuum spectrum of the free space and is completely trapped by a barrier with a finite thickness, we name these states **FBICs** (finite barrier-enabled bound states in the continuum). A FBIC exhibits decaying oscillation inside the PC (potential barrier) which is similar to the original BIC concept proposed in the 1929 paper^40^. In addition, different from BICs on photonic crystal slabs^3^ where the fields are concentrated in the dielectric, the decaying feature of FBICs here ensures that the wavefunctions lay largely inside the air. Such a unique property can further boost light-matter interaction in air based on BICs such as enhancing exciton-photon coupling with 2D layered materials^41^.

## Results

**Fig. 2:**
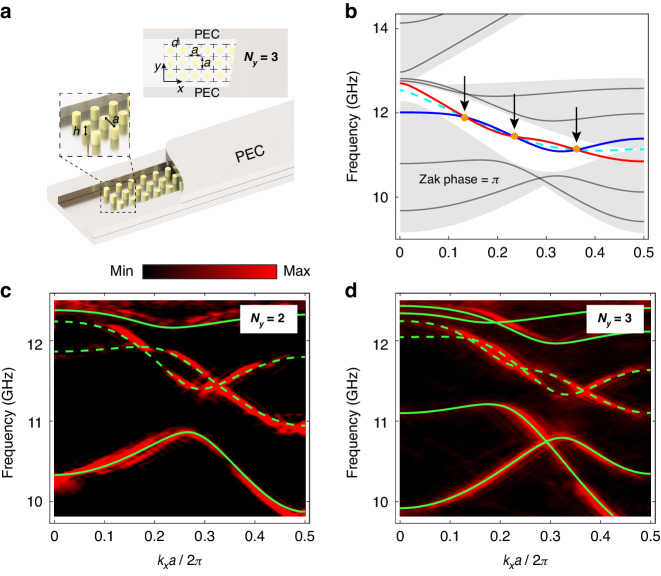
The first configuration, is a strip geometry of a photonic crystal (PC) truncated by PECs on both sides. **a** Illustration of the experimental setup for the case of ${N}_{y}$ = 3. Here faint yellow represents the dielectric cylinders of the PC and light gray denotes the surrounding PECs. **b** The projected band structure (gray) with the dispersion of the boundary mode (cyan) along the ${k}_{x}$ direction when ${N}_{y}$ is large enough. The band structure for ${N}_{y}$ = 3 is plotted with the solid lines, where the gray lines denote bulk bands and the red and blue lines denote the boundary modes. Three nodes are marked by the yellow dots and pointed by the black arrows. **c**, **d** Measured (color code) and simulated (lines) band structures for ${N}_{y}\,$= 2 and 3, respectively. Here the solid green lines represent the bulk modes, and the dashed green lines represent the two boundary modes, respectively. The lattice constant of the PC, the height, radius, and relative permittivity of the cylinder are *a* = 14 mm, *h* = 8 mm, *r* = 3 mm, and ${\varepsilon }_{r}$ = 9.0, respectively. A small air gap *d* = 4 mm is kept between the side PEC boundaries and the PC

Figure 2a sketches our system, where we consider a mirror-symmetrical strip geometry of a square lattice PC truncated by perfect electric conductors (PECs) on both sides. The upper insets show the side and top views. Here faint yellow represents the dielectric cylinders that are embedded in air. Our experiments were conducted in microwave frequency region wherein PECs can be well-approximated by metal plates such as the aluminum plates used in our work. For convenience, the height of the dielectric cylinder is kept small and the PC is sandwiched by two PECs on the upper and lower sides. We consider the TM polarization mode with a uniform electric field pointing out of the plane. Under such a condition, the system in Fig. 2a has an equivalent 2D system^42^.

To start, we first assume the width of the PC as denoted by ${N}_{y}$ is large enough and Fig. 2b shows the band dispersion. Here the gray areas represent the projection of the bulk bands while the cyan dashed line at around 12 GHz denotes the boundary mode. Since this system exhibits mirror symmetry, the dispersions of the boundary mode on both sides of the PC are the same. The presence of the boundary mode has topological reasoning since the Zak phase for the lower band at around 10 GHz is ${\rm{\pi }}$ independent of ${k}_{x}$^43^. We keep a small air gap (*d* = 4 mm) between the side PEC boundaries and the PC such that the dispersion of the boundary mode is deep inside the bandgap. (Supplementary Material Sec. I) When the width of the PC is finite with ${N}_{y}$ being a small integer, the two boundary modes localized on opposite sides of the PC will interact and split into one odd and one even mode with respect to the mirror plane. The solid lines in Fig. 2b show the band dispersion for ${N}_{y}=3$, where the gray lines denote the bulk modes, and the red and blue lines represent boundary modes with even and odd electric field distributions, respectively. The red and blue lines twist with each other and are nondegenerate except for the three nodes as marked by the yellow dots and pointed by the black arrows. At those three nodes, there is no energy splitting and hence the coupling strength vanishes. In other words, the boundary mode localized on one side of the boundary cannot hop to the other side if its wave vector matches one of the nodes. We have numerically confirmed that the number of nodes equals ${N}_{y}$ when it is small. Moreover, the presence of these nodes in our system is robust against parameter variation as long as the mirror symmetry is preserved, see e.g., Fig. S1 where we change the width of the air gap. In fact, the presence of these nodes originated from the dominant orbital components. (See Supplementary Material Sec. II) We emphasize that the number of nodes and ${N}_{y}$ being the same is a unique feature of our system. In Supplementary Material Sec. III, we show that these nodes cannot be found easily in the other bandgap of our PC, or triangular lattice even though the mirror symmetry is preserved.

In the experiment, we excite the sample at one side of the sample, measure the field distributions, and then apply the Fourier transform to obtain their dispersions. We need to move the upper PEC boundary so as to measure the electric field distribution. This experimental setup, however, unavoidably introduces a sub-millimeter (~0.5 mm) air gap between the cylinders and the upper PEC boundary. Such an air gap has limited impacts on the dispersions of the band of interest. (see Supplementary Materials Sec. I). Figure 2c, d show the measured band dispersions (color code) together with the simulated band dispersion (lines) for ${N}_{y}$ = 2 and 3, respectively. (${N}_{y}\,$= 4 and 5 are provided in Fig. S3) The measured dispersion agrees well with the simulations.

**Fig. 3:**
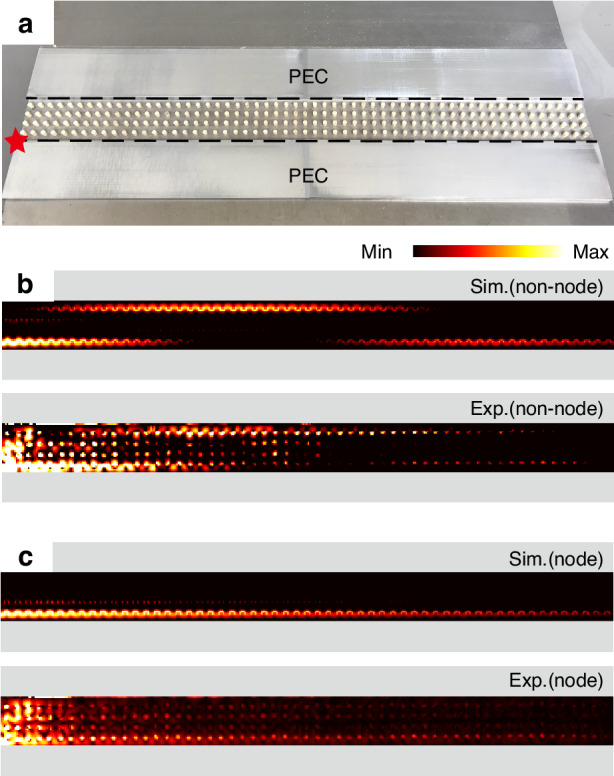
Coupling between boundary modes for *N*_*y*_ = 4 in the first configuration. **a** A photo of the experimental sample with ${N}_{x}\,$= 57, ${N}_{y}\,$= 4, and the red star denotes the position of the point source. **b**, **c** Simulated and experimental electric field distributions for ${N}_{y}\,$= 4 at a non-node frequency of 11.63 GHz, and a node frequency (11.80 GHz in the experiments and 11.82 GHz in the simulations). The relative permittivity of the cylinders is set as ${\varepsilon }_{r}$ = 9.0 + 0.02i

We then proceed to demonstrate that the hopping vanishes for the boundary modes when their wave vector matches the nodes. Figure 3a shows a photo of the experiment setup, and the sample will be covered by another PEC top layer in the experiments and simulations. We fine-tune the source antenna labeled by the red star to excite predominantly the boundary mode localized on the lower boundary. We choose ${N}_{y}\,$= 4 as an example. There are four nodes on the twisting boundary modes and we choose the one at 11.80 GHz (${k}_{x}a/2\pi =0.2$). Meanwhile, we also perform the experiments at 11.63 GHz where the coupling strength is finite. The measured and simulated electric field distributions inside the waveguide are shown in Fig. 3b, c. In the simulations, the relative permittivity of the cylinders is set as ${\varepsilon }_{r}$ = 9.0 + 0.02i with an imaginary part to simulate the inevitable loss in the experiments. As shown in Fig. 3b, at a frequency of 11.63 GHz (not one of the nodal points), the stimulated boundary mode couples to another boundary after propagating tens of lattice sites and then recouples to the original boundary. In contrast, when the frequency matches the frequency of a node (11.80 GHz), the excited boundary mode stays on the lower side while propagating to the right as shown in Fig. 3c. The field oscillation period is inversely proportional to the coupling strength between the boundary modes: the smaller the coupling strength, the longer the oscillation period. The oscillation period is infinite at a node frequency. Thus, just a few lattice sites are able to prohibit the interaction between adjacent boundary guiding modes effectively.

**Fig. 4:**
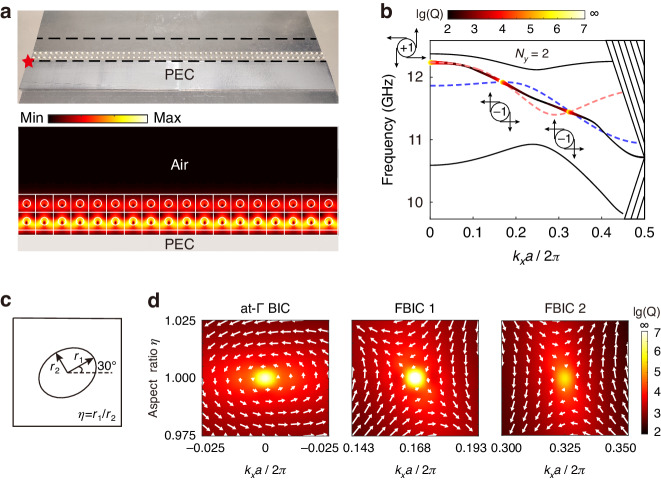
The second configuration, one of the PEC boundaries is replaced by air. **a** The upper panel shows a photo of the sample with ${N}_{y}\,$= 2. The red star denotes the position of the point source. The field inside the region between the black dashed lines was scanned. The lower panel shows the eigenfield amplitude distribution at ${k}_{x}a/2\pi =0.325$ (the second nodal wave vector for ${N}_{y}\,$= 2). **b** The bulk bands (solid black lines), boundary band (colored line), and the corresponding Q factor of boundary mode for ${N}_{y}\,$= 2. **c** The definition of geometric parameter $\eta$ of the tilted elliptical cylinder. Here, the angle between one of the main axes ${r}_{1}$ and the *x*-axis is 30°. The lattice constant is 14 mm and ${r}_{2}$ is fixed at 3 mm. The aspect ratio of the ellipse is defined as $\eta ={r}_{1}/{r}_{2}$. **d** The vector field of the far-field radiation with the Q factor as background around the at-$\Gamma$ BIC and the two FBICs

If we remove the PEC boundary on one side of the PC, there is some probability that the remaining boundary mode can tunnel through the PC and radiate into the free space as the PC is finite. However, if the wave vector matches those of the nodes, the tunneling probability vanishes, and the remaining boundary mode is completely trapped. We call these finite barriers trapped boundary modes FBICs as they also fit into the definition of BICs. The upper panel of Fig. 4a shows the experimental setup, where we replace one of the PEC boundaries with air. The sample will be covered by another PEC top layer in the experiments and simulations. Figure 4b shows the band structure and the Q factor of the remaining boundary mode for ${N}_{y}$ = 2, wherein the colored line marks the boundary band, and the solid black lines denote the bulk bands. The area outside the light cone (lower right shaded region) and above the first-order diffraction limit (upper right shaded region) are not shown. As a reference, the red (even state) and blue (odd state) dashlines denote the boundary mode dispersion for the case with PEC on both sides. The frequencies of the bulk bands and the remaining boundary mode shift slightly. Interestingly, the frequencies of the remaining boundary mode at the nodes, where the red and blue lines cross, do not change. Moreover, besides the usual symmetry-protected BIC at the $\Gamma$ point, another two BICs (FBICs) are emerging at those nodal wavevectors. The lower panel of Fig. 4a shows the amplitude of the boundary mode at the second nodal wave vector (${k}_{x}a/2\pi =0.325$), where we can see the eigenfield vanishes inside the air. More information about the eigenfields is provided in Supplementary Materials Sec. IV. The results for ${N}_{y}\,$= 3 and 4 are provided in Fig. S8, where we can see three and four FBICs, respectively. In short, the remaining boundary mode can be trapped completely at nodal wavevectors within very few lattice sites at which the bulk bandgap is not even well-defined. In contrast, those modes not at nodal wavevectors host a finite Q factor and become leaky modes.

We proceed to show the origin of these FBICs and we start with the topological characterization. Different from BICs in photonic crystal slabs where there are two periodic wavevectors^44^, our system is periodic along only the ${k}_{x}$ direction. We introduce another geometric parameter $\eta$ so as to define a winding number. As shown in Fig. 4c, we change the circular dielectric cylinder to elliptical and $\eta ={r}_{1}/{r}_{2}$ is defined as the ratio between two axes. We tilt the elliptical cylinder such that the angle between one of the main axes ${r}_{1}$ (the long axis when $\eta \,>\, 1$) and the *x* direction is 30°. According to the Bloch theorem, the electric field can be written as ${E}_{{k}_{x},\eta }(x,y)={e}^{i{k}_{x}x}{u}_{{k}_{x},\eta }(x,y)$, where ${k}_{x}$ is the wave vector in the *x* direction, and ${u}_{{k}_{x}}$ is the periodic part of the Bloch wave. Only the TM mode (${E}_{z}$) is considered in our system. The amplitude and phase of the outgoing wave are determined by the zero-order Fourier coefficient $c({k}_{x},\eta )=\left\langle {u}_{{k}_{x},\eta }\right\rangle$, where the bracket $\left\langle \cdot \right\rangle$ denotes the spatial average over a unit cell. In general, the coefficient $c({k}_{x},\eta )$ is complex and we can define a vector field for the far-field radiation as $\vec{c}({k}_{x},\eta )=\mathrm{Re}\left[c({k}_{x},\eta )\right]\widehat{x}+{Im}\left[c({k}_{x},\eta )\right]\widehat{y}$. The topological charge *q* (or the winding number) can be introduced as $q=\frac{1}{2\pi }{\oint }_{C}{dk}\cdot \nabla \phi ({k}_{x},\eta )$, where *C* is a closed simple path in ${k}_{x}-\eta$ parameter space in a counterclockwise direction, and $\phi ({k}_{x},\eta )$ is the phase of $c({k}_{x},\eta )$. The vector field for $c({k}_{x},\eta )$ in the parameter space expanded by ${k}_{x}$ and $\eta$ around the at-$\Gamma$ BIC and these two FBICs is shown in Fig. 4d, wherein the *x* and *y* components of the vectors represent the real and imaginary parts of $c({k}_{x},\eta )$, respectively. The Q factor is also given with the color as the background. The topological charge of the symmetry-protected BIC at the $\Gamma$ point is +1, while those of the two FBICs are both $-1$. Thus, $c({k}_{x},\eta )$ vanishes at the vortex center and the corresponding boundary mode exhibits an infinite Q factor. The topological charges of the three BICs are also sketched in Fig. 4b. The two FBICs sharing the same charge indicate that they cannot be annihilated by each other. We emphasize that the charge we define through the winding number in the $({k}_{x},\eta )$ space does not depend on the tilting angle of the elliptical cylinder. (See Supplementary Materials Sec. IV) Meanwhile, the winding number defined here is different from the Zak phase defined before. The nontrivial Zak phase ensures that the presence of the boundary mode as a function of ${k}_{x}$ while the nonzero winding number here proves that the boundary mode at some special wavevectors, i.e., the nodes, are indeed BICs.

In addition, we also provide an explanation to unveil the underlying physical mechanism of these FBICs. Let us start with the case with PEC on both sides of the PC. The coupling strength between the boundary modes vanishes at these nodes; thus, these two boundary modes degenerate. Such a degeneracy is protected by the mirror symmetry. As a result, an arbitrary linear combination of these two boundary modes at nodes is also an eigenmode of the system when the mirror symmetry is preserved. In other words, the state $\left|\varPsi \right\rangle ={a}_{1}\left|{\psi }_{1}\right\rangle +{a}_{2}\left|{\psi }_{2}\right\rangle$ is also an eigenstate of the system at these nodes, where $\left|{\psi }_{1}\right\rangle$ and $\left|{\psi }_{2}\right\rangle$ represent the boundary modes, and ${a}_{1}$ and ${a}_{2}$ are arbitrary coefficients. As for the case that the PEC boundary is preserved at only one side, one radiation channel is open. The corresponding radiation coefficient is the overlap integral between the modes and the radiated plane wave, i.e., $c=\left\langle {p|}\varPsi \right\rangle ={a}_{1}\left\langle {p|}{\psi }_{1}\right\rangle +{a}_{2}\left\langle {p|}{\psi }_{2}\right\rangle$. The nonradiative condition is reached when *c* = 0. Such a condition can always be satisfied at a specific combination of ${a}_{1}$ and ${a}_{2}$. Thus, there is one “BIC” at each node when only one side of the boundary is PEC. As an alternative explanation, we can consider the field distributions of the eigenmodes. For the case with PEC on both sides, a linear combination of these two boundary modes leads to a new eigenmode whose unit-cell-averaged magnetic field component parallel to the boundary is zero. For such an eigenmode, even if the PEC on one side is removed, it preserves and still cannot radiate to the environment. According to the definition, such a mode should be a “BIC”. Since the field distribution of this mode is mainly localized near the remaining PEC boundary, we name it a finite barrier-enabled BIC.

**Fig. 5:**
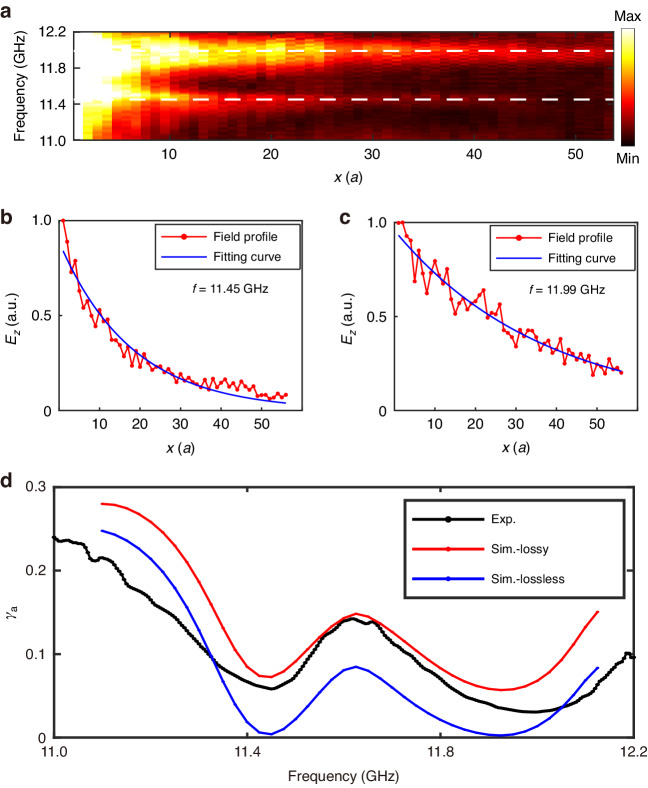
The attenuation of boundary modes in the second configuration. **a** The measured field profiles of the boundary modes at different frequencies. The white dashed lines denote the two FBIC frequencies, which are 11.45 GHz and 11.99 GHz. **b**, **c** The field profiles and the fitting curves at the two FBIC frequencies. **d** The attenuation coefficients $\gamma$ along the boundary lattice obtained from simulations (blue line for the lossless situation and red line for the lossy situation with ${\varepsilon }_{r}$ = 9.0 + 0.02i) and experiments (black line)

The non-radiation feature of FBICs can also be confirmed by transport measurements. We place a point source at the lower-left corner (marked by the red star in Fig. 4a) to excite the remaining boundary mode localized at the PEC boundary. As shown in Fig. 4a, the field of the boundary mode mainly concentrates near the remaining PEC boundary. Thus, for each unit cell along the *x* direction, we average over the field amplitude inside the air gap and the unit cell closest to the PEC boundary and take this value as the field amplitude for that unit cell. Figure 5a shows the measured field profile (average field amplitude inside each unit cell along the *x* direction) of the boundary modes at different frequencies. Near 11.45 GHz and 11.99 GHz (the two experimental FBIC frequencies marked by the white dashed lines), the decay of boundary modes is slower than other adjacent frequencies. The electric fields of boundary modes decay exponentially as a function of *x*, so we fit the field profiles with $A{e}^{-\gamma x}$ and obtain the attenuation coefficient $\gamma$. To clearly show the attenuation of the boundary mode, the field profile and the fitting curve at the two FBIC frequencies are provided in Fig. 5b, c. We can see the fields indeed decay slowest at the FBIC frequencies and the decaying profiles fit well with the function $|{E}_{z}| \sim A{e}^{-\gamma x}$.

The Q factor is inversely proportional to the attenuation coefficients $\gamma$. Since $\gamma$ can be directly extracted from the field distribution, here we use $\gamma$ to calibrate the existence of FBICs. If there is no loss, the boundary mode propagates to infinite; otherwise, $\gamma \,\ne\, 0$. The blue line in Fig. 5d shows the numerically simulated values of $\gamma$ when we ignore the intrinsic material loss. Here we focus on the frequency range where there is only the boundary mode and hence the influence from other bulk modes can be safely ignored. $\gamma$ are zero at the FBICs frequencies 11.45 GHz and 11.92 GHz (For this FBIC, the frequency is 11.92 GHz numerically and 11.99 GHz experimentally, with slight differences due to sample imperfections). $\gamma$ is finite when the working frequency deviates from these two frequencies. In the experiments, there is unavoidable material loss, and the total $\gamma$ is given by ${\gamma }_{{tot}}={\gamma }_{{rad}}+{\gamma }_{{abs}}$, where ${\gamma }_{{rad}}$ and ${\gamma }_{{abs}}$ represent the attenuation when considering only the radiation loss and absorption loss. In the simulations, the relative permittivity of the cylinders is set as ${\varepsilon }_{r}$ = 9.0 + 0.02i. The red line in Fig. 5d shows the simulated $\gamma$ when the absorption loss is considered. Comparing the red and blue lines in Fig. 5d, we can see that ${\gamma }_{{abs}}$ varies only a little in the interested frequency range. Thus $\gamma$ as a function of frequency exhibits two dips at the two FBICs frequencies. Such a feature has also been confirmed experimentally as shown with the black line in Fig. 5d. The deviation of the black line from the red line might be due to sample imperfections. As a supplement, we provide the measured field distribution at a non-FBIC frequency and the two FBIC frequencies (see Fig. S10 in Supplementary Materials Sec. V).

## Discussion

### Summary

We provide two typical configurations and demonstrate experimentally how a state is trapped by a finite barrier. The first configuration offers a feasible approach for fine-tuning the hopping of trapped states, which is a crucial ingredient for nanophononics such as non-abelian braiding of photons^16^ and integration of multiple waveguides into a compact device^17^. The second configuration provides a new mechanism for realizing BICs. Instead of being concentrated inside the PC, the field of an FBIC is outside of the PC, which can thus boost light-matter interaction schemes based on BICs. This physics discussed in our work is general and can be extended into other wave systems such as phononic crystals and cold atoms.

## Materials and methods

### Theory and simulation

The numerical band structure, the electric field distributions under source antenna excitation and the vortex of far-field radiation shown in Figs. 2, 3, and 4 were obtained by using commercial software COMSOL Multiphysics^45^.

### Experimental setup

The PC consisting of an alumina rod array was fixed to the bottom metal (aluminum) plate. The microwave was radiated from an antenna connected to our vector network analyzer (VNA, Keysight N5242B), and electric field distribution was measured by an antenna (also connected to the VNA) fixed in a hole in the upper metal plate. The relative position of the field-scanning antenna with respect to the PC was controlled by a stepping motor.

### Supplementary information

supplementary materials for Finite barrier-bound state
