# Supplementary material for: Finite barrier bound state
Source: Light Sci Appl. 2024 Mar 8;13:69. doi: 10.1038/s41377-024-01417-1 (PMC10920789; doi:10.1038/s41377-024-01417-1)
Supplement: Supplementary file 1 — supplementary materials for Finite barrier-bound state [file 41377_2024_1417_MOESM1_ESM.pdf]

Supplementary Information for

"Finite barrier bound state"

Tao Liu[1], Kai Bai[1], Yicheng Zhang[1], Duanduan Wan[1†], Yun Lai[2], C.T. Chan[3], and Meng Xiao[1, 4*]

[1]Key Laboratory of Artificial Micro- and Nano-structures of Ministry of Education and School of Physics and Technology, Wuhan University, Wuhan 430072, China

[2]National Laboratory of Solid State Microstructures, School of Physics, and Collaborative Innovation Center of Advanced Microstructures, Nanjing University, Nanjing 210093, China

[3]Department of Physics, The Hong Kong University of Science and Technology, Clear Water Bay, Kowloon, Hong Kong, China

[4]Wuhan Institute of Quantum Technology, Wuhan 430206, China

Corresponding E-mail: † ddwan@whu.edu.cn; * phmxiao@whu.edu.cn

This PDF file includes:

I. The influence of the air gaps

II. Dependence of the twisting of the boundary modes on the orbitals

III. Boundary modes without nodes

IV. The eigenfields and the vortex characteristic of FBICs

V. The field distributions of boundary modes in the second configuration

Fig. S1-S10

References

I. The influence of the air gaps

In this section, we discuss the influence of dielectric cylinders' height and the air gap in our experimental setups. The experimental setup is 3D and one needs the top and bottom PEC boundaries to enforce that the electric field is along the z-direction. For experimental convenience, we chose the height of dielectric cylinders to be h = 8 mm which introduces higher order modes along the z direction. Besides, there are two possible air gaps in our experiments. First, since we need to move the top PEC boundary so as to measure the field distributions, there is an unavoidable top air gap between the cylinder and the top PEC boundary. Second, to better characterize the dispersion and field distribution of the boundary modes, we also need a side air gap between the boundary of the PC and the side PEC.

We start with the effects of the side air gap, whose width (denoted by d) is defined in Fig. S1a. Same as the main text, we consider the band structure of a 2D PC with the TM polarization (electric field along the z direction). The PC has a lattice constant a = 14 mm, and the radius and the relative permittivity of cylinders are set as r = 3 mm and ε_r = 9.0, respectively. When the width of the PC as denoted by N_y is large enough, the band dispersion under three different widths of the side air gap d is shown in Figs. S1 b-d. Here the gray areas represent the projection of the bulk bands while the dashed cyan line denotes the boundary mode. As the width of the side air gap increases, the frequencies of boundary modes (cyan dashed line) gradually decrease and evolve from the upper boundary to the lower boundary of the band gap.[1] Then we keep $N_y = 3$ and consider these three typical values of d as plotted in Figs. S1b-d. Same as before, gray lines for the bulk modes, the red and blue lines for the even and odd boundary modes of finite PCs. When the air gap length varies gradually, the number of nodes keeps unchanged while the corresponding k_xs shift. We can see that such a peculiar twisting feature is robust even if the width of the side air gap varies within a reasonably large range.

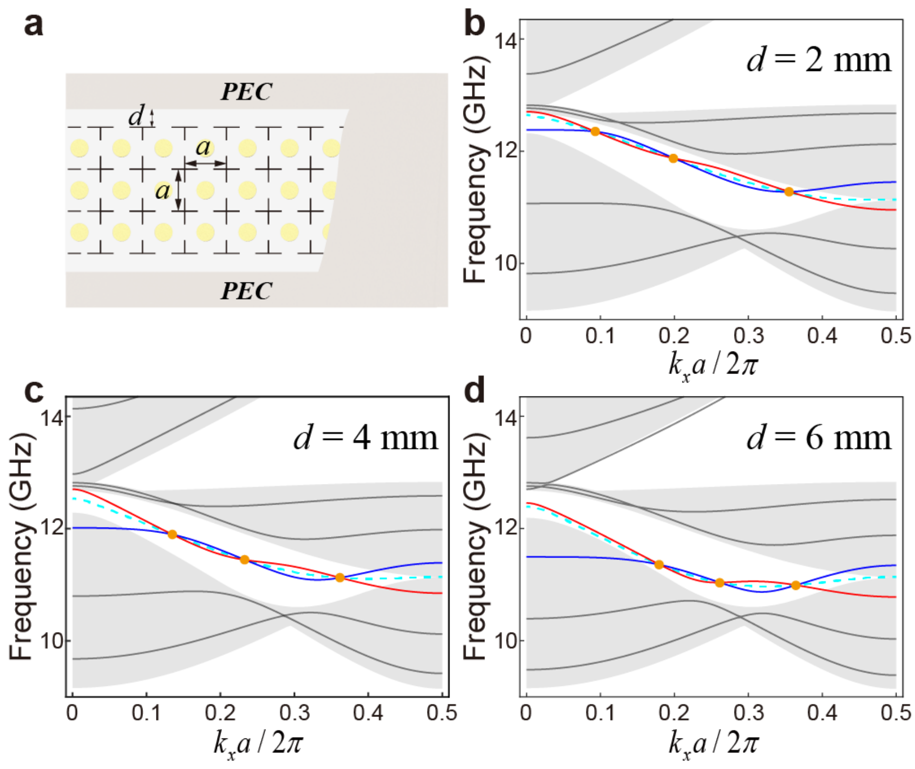

Figure S1 | The influence of the side air gap. a, Sketch shows the definition of the side air gap, where the lattice constant is a = 14 mm. **b-d,** The projected band structure (gray) with the dispersion of the boundary mode (cyan) along the k_x direction when N_y is large enough under three different widths of the side air gap. The solid lines represent the band structures for $N_y = 3$, where the gray lines denote bulk bands and the red and blue lines denote the boundary modes. The height, radius and relative permittivity of the cylinder are h = 8 mm, r = 3 mm, and ε_r = 9.0, respectively.

Then we proceed to consider the effect of the top air gap. Figure S2a shows the band structure of a 2D PC as considered in Fig. S1. In the experiments, dielectric cylinders of height 8 mm are sandwiched by the top and bottom PEC layers. The black dashed line in Fig. S2b shows the corresponding band dispersion. Compared with Fig. S2a, there are three additional flat bands with two nearly degenerate bands at around 12.5 GHz and one at around 15 GHz. The eigenfields of these flat bands exhibit a sinusoidal dependence along the z direction (higher-order modes) with fields dominantly localized inside the dielectric cylinders. Since mirror symmetry is still preserved here, these higher-order modes do not interact with the fundamental modes. Meanwhile, these higher-order modes can be shifted to a higher frequency by

decreasing the height of the dielectric cylinders. In our experimental setup, there is always a sub-millimeter air layer between the top of the dielectric cylinders and the PEC. Such an air gap breaks the up-down mirror symmetry and introduces coupling between the nearly flat higher-order modes and fundamental modes. The red lines in Fig. S2b represent the band dispersion when there is a 0.5 mm air gap. Figure S2c shows a few typical eigenmodes as marked in Fig. S2b. Here A1 and A2 are higher-order modes that exhibit dipole-like in-plane field distributions, B, D1 and D2 are fundamental dipole modes, and C is a monopole. For the bands of interest, the air gap decreases the effective relative permittivity of the dielectric cylinders while the nodes are still preserved as can be seen in Figs. 2c,d in the main text.

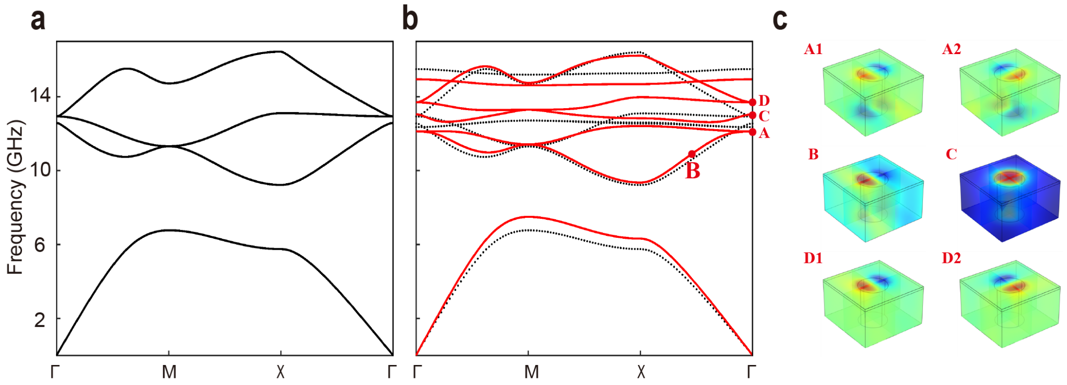

Figure S2 | The influence of the top air gap. a, The band structure of a 2D PC. **b,** The black dashed lines and the solid red line represent the band structure of 3D PCs when there are no air gap and a 0.5 mm air gap, respectively. **c,** The electric field distribution of a few representative eigenmodes [marked in **b**]. The relative permittivity of the cylinders is set as ε_r = 8.6 for **a** and the black dashed lines in **b**, and ε_r = 9.0 for the solid red lines in **b**. The height of the dielectric cylinders is 8 mm in **b**. Except for the parameters mentioned above, all the other parameters used are the same as in Fig. 2 in the main text.

Considering the presence of the top air gap in the experiments, we introduce a tiny air gap between the cylinders and the top PEC in the simulations to fit the experimental results. Here we provide the measured band dispersions (color code) together with the simulated band dispersion (lines) for N_y=2, 3, 4 and 5 in Fig. S3. Obviously, this particular type of boundary mode twisting persists even if the sub-millimeter air gap is introduced and the measured dispersion agrees well with the simulations.

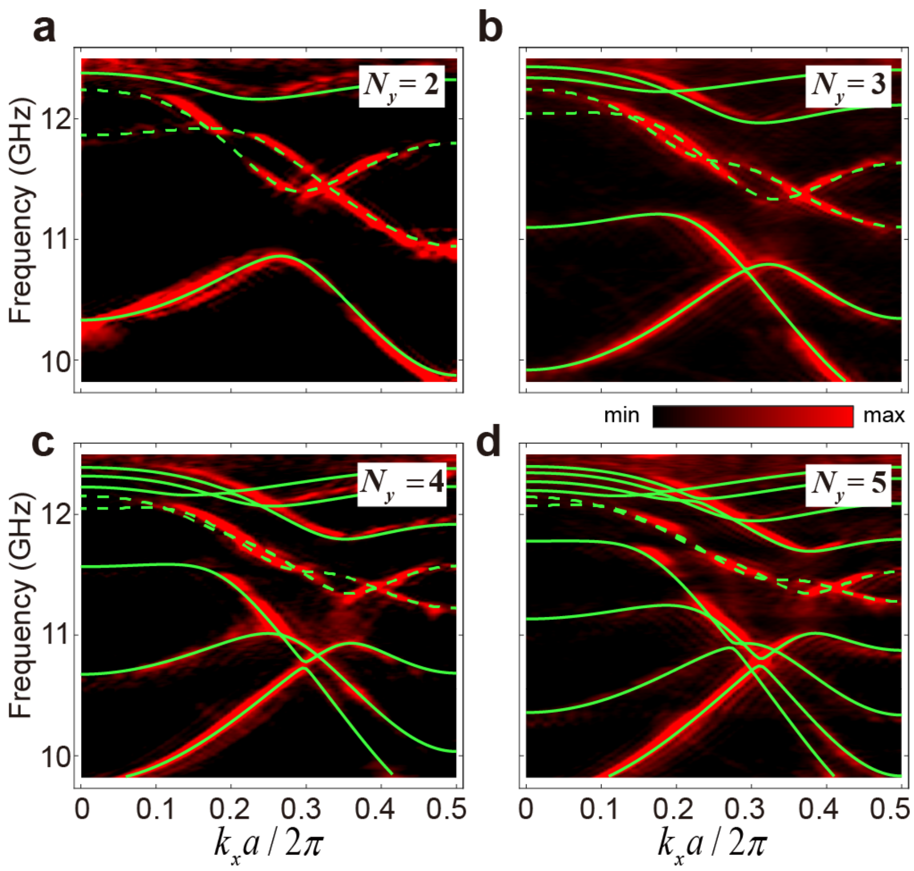

Figure S3 | Measured (color code) and simulated (lines) band structures for N_y =2, 3, 4, 5, respectively. Here the solid green lines represent bulk modes, and the dashed green line for the two boundary modes, respectively. In the simulations, a tiny air gap of thickness 0.4 mm, 0.3 mm, 0.5 mm, 0.5 mm between the cylinders and the top PEC cover is introduced for N_y =2, 3, 4, 5, respectively. The number of lattice sites in the *x*-direction is kept at N_x = 57 which is enough to map out the mode dispersions. The lattice constant of the PC, the height, radius and relative permittivity of the cylinder are a = 14 mm, h = 8 mm, r = 3 mm, and ε_r = 9.0, respectively. A small air gap d = 4 mm is kept between the side PEC boundaries and the PC.

II. Dependence of the twisting of the boundary modes on the orbitals

In Ref. [2], we have proposed a special type of finite size effect (FSE) for some waveguide modes that apparently vanishes at some particular wave vectors along the waveguide. Meanwhile, the number of wave

vectors where the FSE vanishes equals the number of lattice sites across the strip. We have also provided a tight-binding model Hamiltonian which shows this peculiar feature based on the coupled in-plane dipoles P_x and P_y. As shown below, this special FSE-induced twisting boundary modes with nodes also exists in PCs with P_x and P_y modes in a square lattice.

Previous studies showed that for a 2D PC consisting of dielectric cylinders in air and considering the polarization with the TM polarization, the typical three lowest order modes are one monopole M_z and two in-plane dipoles P_x and P_y [3]. Specifically, we give a 2D PC where the P_x and P_y orbitals are far away from the M_z orbital, i.e., almost no orbital hybridization with the M_z orbital. This condition is achieved when the frequency of the singly-degenerated state (M_z) is higher than the doubly-degenerated states (P_x and P_y) at the Γ point. We consider a 2D square lattice PC with a lattice constant a = 12 mm, and the radius and the relative permittivity of the cylinders are r = 3 mm and ε_r= 9.0, respectively. Figure S4a shows the simulated band structure. The frequency of the singly-degenerated state (14.5GHz) is higher than that of the doubly-degenerated states (13.1GHz) at the Γ point. Zak phase of the second band as a function of k_x keeps π, which indicates the existence of a boundary mode as shown in Fig. S4b with the cyan line. The band gap closes only at $k_x = 0$ and $k_x = \pi / a$, and the boundary modes connect these two bands closing points inside the band gap.

Now we proceed to a finite system of strip geometry which is periodic along the x-direction and finite along the y-direction, and the number of lattice sites along the y-direction is N_y. Here we show cases N_y = 2, 3, 4 and 5 as examples to demonstrate the peculiar feature that we focused on. We have also checked the higher N_y cases. The band dispersions for N_y= 2, 3, 4 and 5 are shown with the lines in Fig. S4c-f, respectively. Here the projected band structures are given by the light gray background as a reference; the black lines and colored lines (red or blue) represent modes with fields predominately at the bulk and the boundary, respectively. Red represents the even modes for the electric field and blue denotes the odd modes. In Fig. S4c-f, it is clear that the number of nodes equals the number of lattice sites N_y. In other

words, such a 2D PC also exhibits these peculiar features as presented by the tight-binding model in Ref. [2].

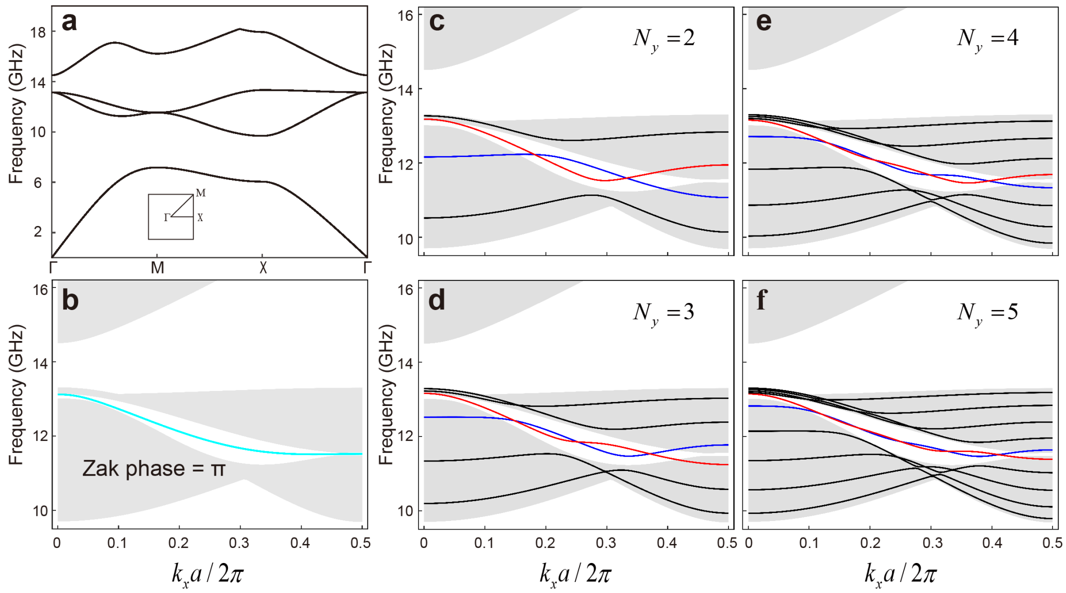

Figure S4 | Twisting of boundary modes with minor hybridization of the M_z orbital. a, The bulk band structure of a 2D PC. The inset shows the reciprocal space. **b,** The projected band structure along the k_x direction, where the gray backgrounds represent the projection of the bulk bands, and the cyan line represents the boundary mode dispersion. **c-f,** The band structures (solid lines) for N_y = 2, 3, 4 and 5, respectively. Here the gray background is the projected band structure in **b**, the black lines represent bulk modes, and the red and blue lines represent boundary modes with even and odd electric field distributions, respectively. In the simulations, the lattice constant is a = 12 mm, and the radius and the relative permittivity of the cylinders are r = 3 mm and ε_r = 9.0, respectively.

Furthermore, the vanishing of coupling is robust even if the monopole mode is hybridized with the two in-plane dipole modes. The reason lies in the fact that the nodes between the odd and even boundary modes are protected by the mirror symmetry and the hybridization with M_z does not break this mirror symmetry. Such a feature then further releases the constraints on the observation. In the main text, we provide an experimental demonstration within a 2D dielectric PC with a lattice constant a = 14 mm in the microwave regime in the presence of hybridization with M_z, where the frequencies of the monopole

mode and the dipole modes at the Γ point are 12.3 GHz and 12.7 GHz, respectively, and thus the lower two bands are not pure in-plane dipole modes.

III. Boundary modes without nodes

In this section, we show that the presence of nodes on the boundary modes is nontrivial. Firstly, we show that by changing the dominated orbital, there is no node on the boundary modes. We still use the same PC. That is: the PC has a lattice constant *a* = 14 mm, and the radius and the relative permittivity of cylinders are set as *r* = 3 mm and ε_r = 9.0, respectively. We can define half of the frequency difference between the even and odd boundary modes to characterize the magnitude of coupling, i.e., $\Delta f = (f_e - f_o)/2$, where f_e and f_o are the frequency of the even mode and the odd mode, respectively. When $\Delta f = 0$, there is no coupling between the two boundary modes, which thus corresponds to a node. In Fig. S5a, we plot Δf for Figs. S3a-d as an example. We can see that Δf crosses zero N_y times indicating there are N_y nodes on the boundary bands.

Now we consider the boundary mode inside a different band gap at a lower frequency range. To properly control the dispersion of the boundary mode, we move the side PEC boundary away from the PC by *d* = 14 mm [a lattice constant, see the inset in Fig. S5b] to create a defect boundary mode. The dispersion of this defect boundary mode is shown in Fig. S5b with the magenta line, where the gray regions also represent the projection of the bulk modes. The boundary mode merges into the bulk band at $k_x = 0.69\pi / a$. Figure S5c shows Δf for N_y = 2, 3, 4, and 5 as functions of k_x for these two boundary modes. Here the black dashed line marks the value of k_x where the boundary mode merges into the bulk. The modes on the right side of this black line are no longer boundary modes. The frequency difference defined here for $k_x > 0.69\pi / a$ is between the two bands which continuously connect the boundary modes for a finite N_y. We can see that away from this black dashed line to the left, the sign of Δf versus k_x does not change for each N_y. Near the black dashed line, Δf crosses zero for N_y = 3 and

5 where the boundary modes interact with the bulk modes and thus the coupling is no longer well defined. In a short summary, the coupling here for the trivial case does not vanish for any specific k_x values.

Figure S5d shows the measured band dispersion (color code) together with the simulated band dispersion (lines) for the case of $N_y = 2$. Here the third and fourth bands (dashed green line) correspond to the boundary modes which do not cross for all the k_xs. Meanwhile, we can still see the crossing of the sixth and seventh bands (vanishing of the coupling coefficient) which are the boundary modes induced by the P_x and P_y orbitals. The measured dispersion agrees well with the simulations.

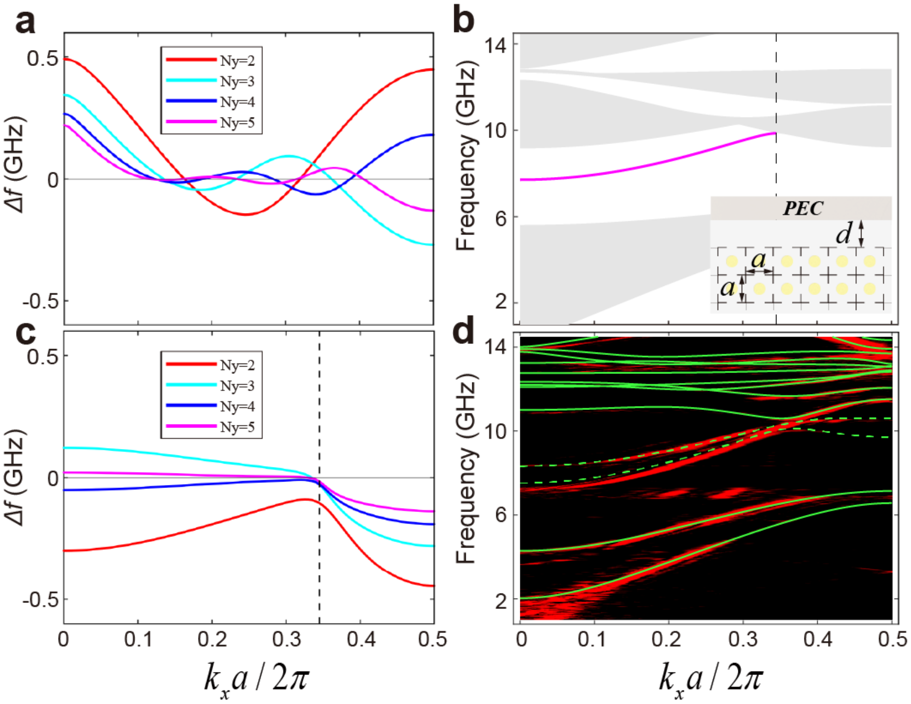

Figure S5 | Trivial case of boundary mode without twisting feature inside a different bandgap in a 2D square lattice PC. a, Δf as functions of k_x for different N_ys. **b,** A defective boundary mode is introduced by shifting the side PEC boundary. Here the magenta line represents the dispersion of the defective boundary mode and the gray regions denote the projection of the periodic bulk bands. The inset sketches the details of the boundary condition. **c,** Δf as functions of k_x for different N_y for the defective boundary mode considered in **b**. The black dashed line marks the position whereat the boundary mode merges into the bulk. **d,** Measured (color code) and simulated (lines) band structures with $N_y = 2$.

Here the solid green lines represent bulk modes, and the dashed green line for two boundary modes, respectively. The parameters of the PC are the same as in Fig. 2 in the main text. In **d**, a 0.5 mm air gap between the cylinders and the upper PEC boundary is introduced in the simulation.

Then we proceed to show that when changing from square lattice to triangular lattice, the nodes of the boundary mode disappear once again. For comparison, we still focus on the gap formed between the P_x and P_y orbitals. We consider a 2D triangular lattice PC with a lattice constant a = 14 mm, and the radius and the relative permittivity of the dielectric cylinders are r = 3 mm and ε_r= 9.0, respectively. The bulk band structure of a 2D triangular lattice PC is shown in Fig. S6a. The insets show the reciprocal space and the eigenmodes of the second and third bands at the Γ point. Similarly, the P_x and P_y orbitals are at around 11.5 GHz and far away from the M_z orbital in such a PC. To construct the boundary mode, we consider a strip geometry structure with mirror symmetry as shown in Fig. S6b. The lattice number along the y direction N_y is kept to be odd in order to preserve the mirror symmetry. The strip of PC is periodic along the x direction and sandwiched by two PECs on the upper and lower sides. The distance between the side PEC boundaries and the center of the boundary lattice is $d = \sqrt{3}a$. We start with the case when N_y is large enough, and correspondingly the interaction between two boundary modes is negligibly small. The band dispersion is shown in Fig. S6c, where the cyan line at around 11 GHz denotes the boundary mode dispersion and the gray areas represent the projection of the bulk bands of the P_x and P_y orbitals. When the width of the PC is finite with N_y being a small number, the two boundary modes localized on opposite sides of the PC form one odd and one even mode with respect to the mirror plane. As shown in Fig. S6d-f, the even (red) and odd (blue) boundary modes dispersion do not cross within the gap, although such boundary modes still originate from the P_x and P_y orbitals and the mirror symmetry is preserved. The twisting feature of boundary mode dispersion is nontrivial.

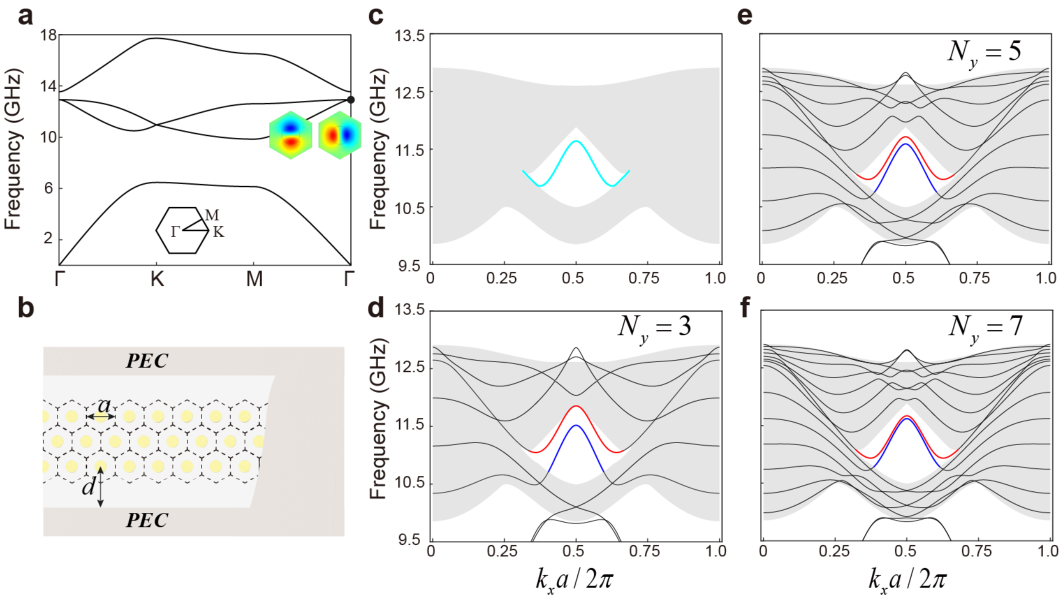

Figure S6 | Trivial case of boundary mode without twisting feature inside P_x and P_y orbitals bandgap in a 2D triangular lattice PC. a, The bulk band structure of a 2D triangular lattice PC, the insets show the reciprocal space and the eigenmodes (E_z field) of the second and third bands at the Γ point. **b,** The sketch of a strip geometry of a 2D triangular lattice PC of $N_y = 3$ with mirror symmetry. **c,** The projected band structure along the k_x direction, where the gray backgrounds represent the projection of the bulk bands, and the cyan line represents the boundary mode dispersion. **d-f,** The band structures (solid lines) for $N_y = 3$, 5 and 7, respectively. Here, the gray background is the projected band structure in **c**, the black lines represent bulk modes, and the red and blue lines represent boundary modes with even and odd electric field distributions, respectively.

IV. The eigenfields and the vortex characteristic of FBICs

In this section, we discuss the eigenfield distributions and Q factor of the remaining boundary modes, and confirm the vortex characteristic of FBICs in the $k_x - \eta$ parameter space. When one of the PEC boundaries is removed, the remaining boundary mode at these nodal wave vectors is completely trapped and exhibits an infinite Q factor, namely FBICs. Thus, the number of FBICs equals the number of nodes.

The eigenfield distributions of the remaining boundary modes at one of the nodal wave vectors and away from the nodal wave vectors are shown in Fig. S7. Here the parameters used are the same as in Fig. 4. When one of the PEC boundaries is replaced by air, the frequencies of the boundary mode (except for those at nodes) near the remaining PEC boundary shift slightly. Meanwhile, the boundary modes at the nodal wave vectors are completely trapped (FBICs) and thus exhibit an infinite Q factor, while those modes not at the nodal wave vectors tunnel through and become leaky modes. Figures S7a and S7b show the real and imaginary parts of the eigenfield distribution at the first nodal wave vector ($k_x a/2\pi = 0.168$) for $N_y = 2$. It is clear that the eigenfield of the remaining boundary mode vanishes entirely inside the air. In contrast, the eigenfield at a non-nodal wave vector ($k_x a/2\pi = 0.25$) leaks into the air, as shown in Figs. S7c and S7d.

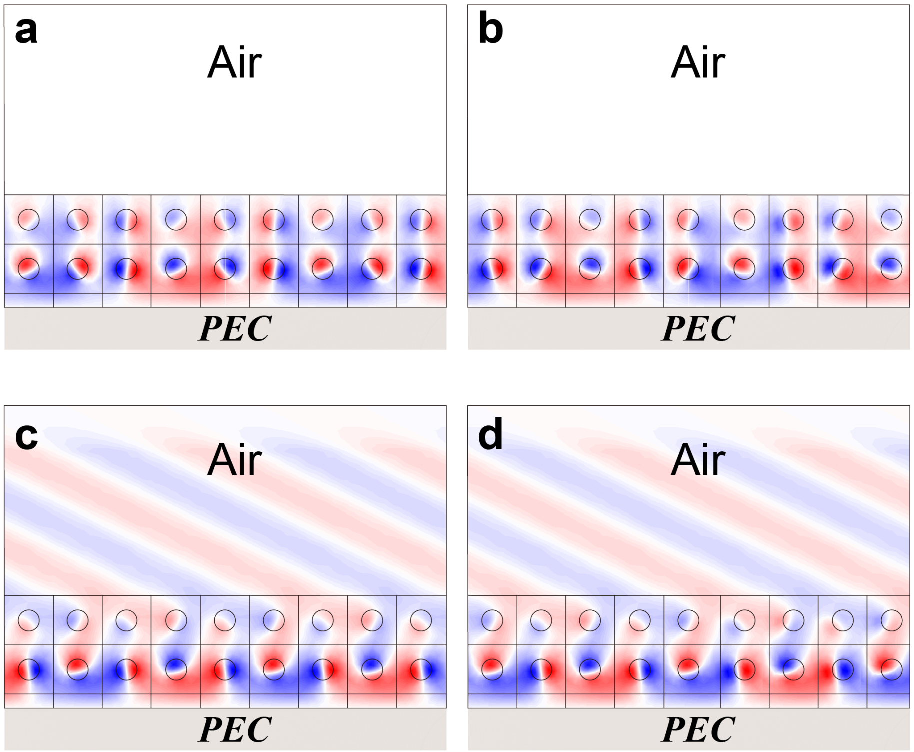

Figure S7 | The eigenfield distribution of the boundary mode for $N_y = 2$. a,b The real and imaginary parts of the eigenfield distribution at the first nodal wave vector in Fig. 4b ($k_x a/2\pi = 0.168$). **c,d** The real and imaginary parts of the eigenfield distribution at a non-nodal wave vector ($k_x a/2\pi = 0.25$). All the parameters used are the same as Fig. 4 of the main text.

Next, the band structure and the Q factor of the remaining boundary mode for N_y = 3 and 4 are shown in Fig. S8. The colored line marks the boundary band and the solid black lines denote the bulk bands. As a reference, the red (even state) and blue (odd state) dash lines denote the boundary mode dispersion in Figs. S3b and S3c. The remaining boundary modes at these nodal wave vectors (FBICs) exhibit an infinite Q factor. Noting that these are also symmetric protected BICs at the Γ point.

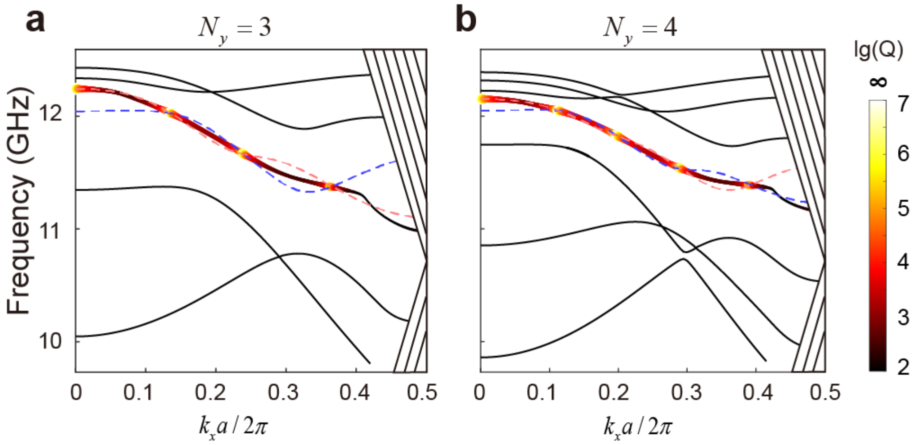

Figure S8 | Infinite Q factor at these nodel wave vectors for N_y = 3 and 4. The bulk bands (solid black lines), boundary mode (colored lines), and the corresponding Q factor of the remaining boundary mode. Except for the one-side open boundary, all the other parameters are the same as those in Fig. S3b and S3c.

At last, we show the vortex characteristic of FBICs in the $k_x - \eta$ parameter space. The presence of a vortex proves that the boundary modes at those nodal wave vectors are genuine BICs instead of high-Q resonances. For the configuration in Fig. 2 and 3 of the main text, the tunneling probability for the boundary modes vanishes at these nodes and therefore the boundary modes do not couple with each other. This feature is protected by the mirror symmetry with which the odd and even boundary modes do not couple. If we replace the circular dielectric cylinders with tilting elliptic dielectric cylinders, the mirror symmetry in the y direction is broken, and there are no longer any nodes. Then if one of the PEC boundaries is replaced with air, the boundary mode at nodal wave vectors will exhibit a finite Q factor and leak to the air environment. Here the tilting angle θ is defined as the angle between one of the main axes r_1 (the long axis when $\eta > 1$) and the x direction, whose range is restricted to 0° $< \theta <$ 90°. The

aspect ratio η of the ellipse (the ratio between two axes of the ellipse, $\eta = r_1 / r_2$) can be a tunable parameter at a certain tilting angle, say, θ = 30°, to tune the Q factor of these states.

In the main text, we define a vector field consisting of the far-field radiation of the electric field in the $k_x - \eta$ parameter space and show the vortex characteristic of FBICs under θ = 30°. The topological charge of the symmetry-protected BIC at the Γ point is +1, while those of the two FBICs are both -1. Here, we show that the topological charge in the $k_x - \eta$ parameter space does not depend on the tilting angle of the elliptical cylinder. To confirm this feature, the vortex characteristic of FBICs with the tilting angles θ = 45° and θ = 60° are presented in Fig. S9. Obviously, the topological charge of all BIC remains the same under different tilting angles.

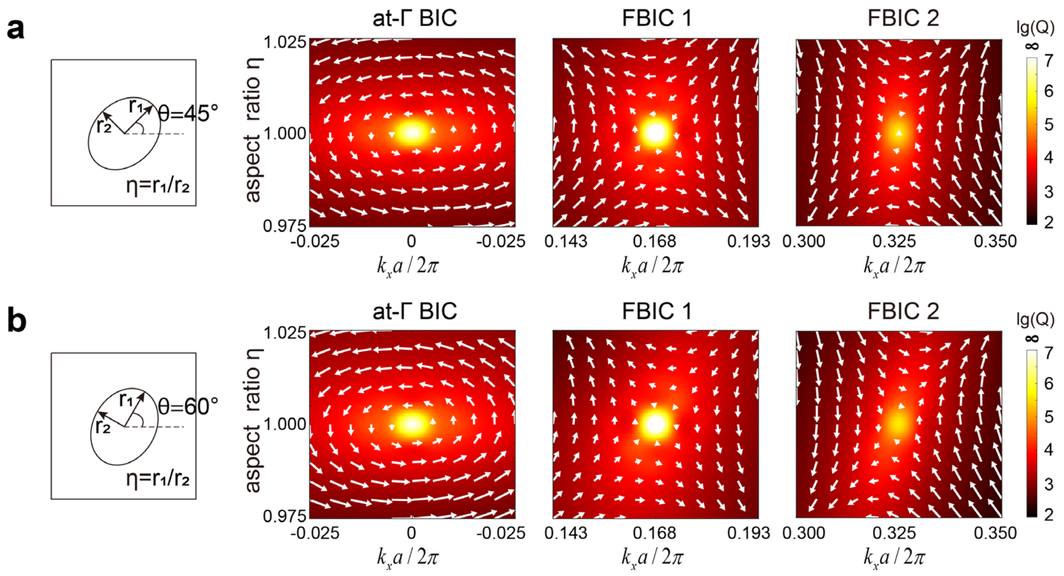

Figure S9 | The geometry configurations of the unitcell and the vortex characteristic of the at-Γ BIC and the two FBICs in the $k_x - \eta$ parameter space for $N_y = 2$. **a,** The tilting angle is θ = 45°. **b,** The tilting angle is θ = 60°.

V. The field distributions of boundary modes in the second configuration

In this section, we show the field distributions at both a non-FBIC frequency and two FBIC frequencies for N_y=2 when the PEC on one side is removed. At non-FBIC frequencies (11.63GHz in both the simulation and experiment), the boundary mode dramatically decays along the boundary direction and propagates for only several lattice sites. In contrast, the boundary mode at FBIC1 frequency (11.92 GHz in the simulations and 11.99 GHz in the experiments) and FBIC2 frequency (11.45GHz in both the simulation and experiment) propagates for quite a few lattice sites before decaying out due to inevitable loss.

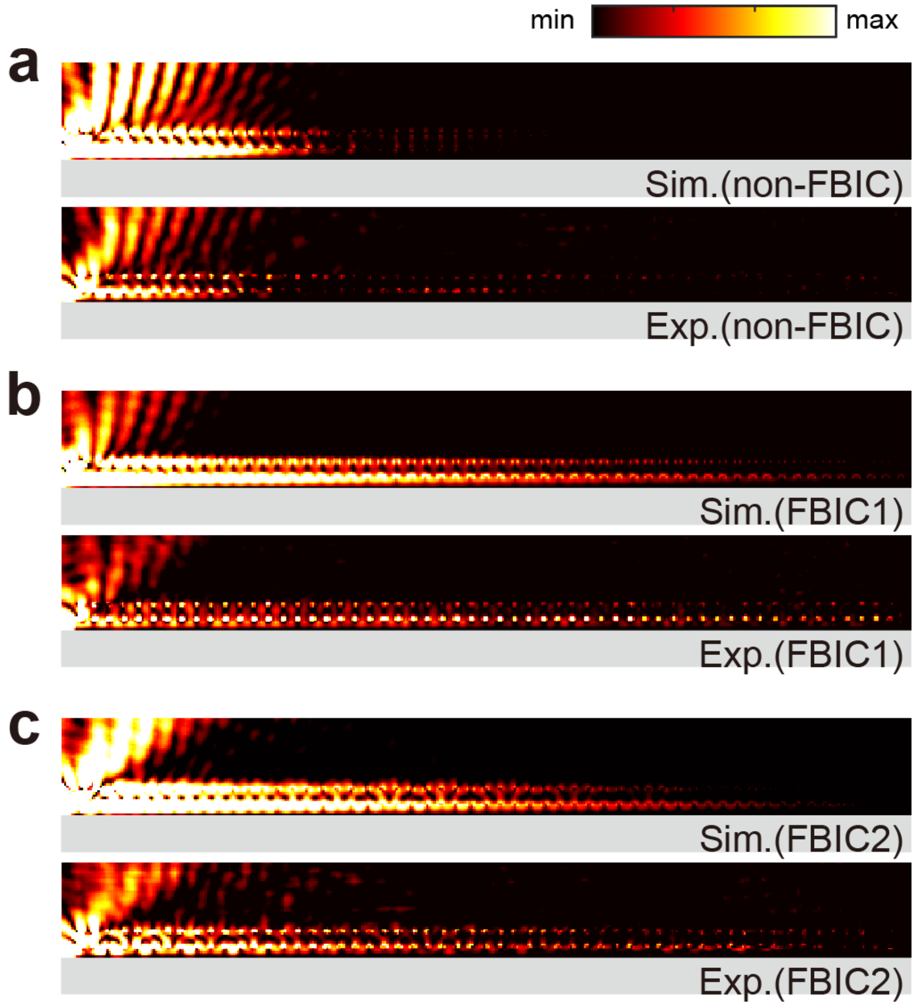

Figure S10 | The electric field distributions at a non-FBIC frequency and two FBIC frequencies for N_y=2. Except for the one-side open boundary, all the other parameters used are the same as in Fig. 2 of the main text.

References:

1. Huang, X. et al. Sufficient condition for the existence of interface states in some two-dimensional photonic crystals. *Physical Review B* **90,** 075423 (2014).
2. Liu, T. et al. The suppression of Finite Size Effect within a Few Lattices. *New Journal of Physics* (Accepted 6 February 2024) In press https://doi.org/10.1088/1367-2630/ad26bb.
3. Huang, X. et al. Dirac cones induced by accidental degeneracy in photonic crystals and zero-refractive-index materials. *Nature Materials* **10,** 582-586 (2011).
